# A Case of Severe COVID-19 in a Patient with Acute Graft-versus-Host Disease after Haploidentical Transplantation

**DOI:** 10.1155/2021/8878803

**Published:** 2021-03-17

**Authors:** Dan Han, Yuqian Sun, Rong Xie, Xiaojian Zhu, Zhaodong Zhong

**Affiliations:** ^1^Department of Hematology, Union Hospital, Tongji Medical College, Huazhong University of Science and Technology, Wuhan 430022, China; ^2^Peking University Institute of Hematology, Peking University People's Hospital, Beijing Key Laboratory of Hematopoietic Stem Cell Transplantation, Beijing 100044, China; ^3^Department of Hematology, Tongji Hospital, Tongji Medical College, Huazhong University of Science and Technology, Wuhan 430030, China

## Abstract

We report a case of coronavirus disease 2019 (COVID-19) after haploidentical transplantation with acute graft-versus-host disease (aGVHD). COVID-19 and aGVHD were improved under treatment with arbidol, remdesivir, methylprednisolone, and ruxolitinib. However, eventually, the patient died of septic shock and multiple organ failure. It was concluded that the disease condition of this COVID-19 patient after transplantation was serious, complex, and variable, with poor prognosis.

## 1. Introduction

Coronavirus disease 2019 (COVID-19) is a respiratory disease caused by severe acute respiratory syndrome coronavirus 2 (SARS-CoV-2). SARS-CoV-2 is highly contagious, and the population is generally susceptible to it. In China, the incidence of COVID-19 in the general population is 5.76/100 000, of which 81% are mild, 14% are severe, and 5% are critical cases; the mortality is 2.3% [1]. In addition, nearly 60% of infected individuals are asymptomatic [2].

Allogeneic haematopoietic stem cell transplantation (allo-HSCT) is an effective treatment for haematological malignancy. However, the risk of respiratory tract infection is higher because of the persistent immunodeficient state after transplantation. Graft-versus-host disease (GVHD) is an immune disease caused by graft-versus-host reaction, which is a serious complication and the main cause of death in allo-HSCT. We diagnosed and treated a COVID-19 patient with aGVHD after haploidentical transplantation.

## 2. Case Presentation

**Figure 1:**
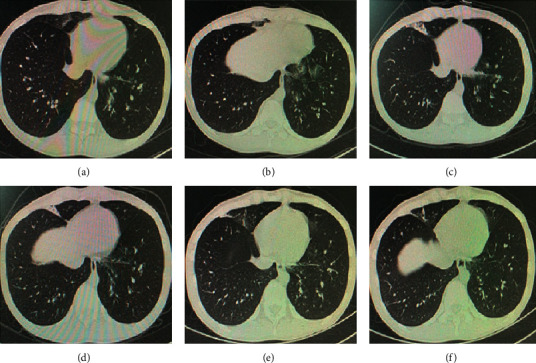
Chest imaging results of the patient during hospitalization. (a, b) (Day 2) Scattered bilateral high-density shadows and ground-glass opacities in the lungs. (c, d) (Day 12) Blurred and patchy shadows scattered in the upper and middle lobes of the right lung and the lower lobe of both lungs. (e, f) (Day 24) Bilateral patchy shadows and filamentous shadows in the lungs, and ground-glass opacity in the lower lobes was slightly increased.

A 20-year-old male patient was admitted to the hospital for “cough and expectoration for 3 days”. He had a history of acute B lymphoblastic leukemia and received peripheral blood stem cell transplantation one year ago. Three days prior, skin biopsy of his left upper arm mass confirmed extramedullary recurrence. Laboratory test results are shown in Table 1. Chest CT (Figures 1(a) and 1(b)) showed bilateral scattered high-density shadows and ground-glass opacities in the lungs. Day 3 (on the third day of admission): he received chemotherapy with cytarabine, decitabine, and etoposide. Day 9: transfusions of G-CSF-mobilized peripheral mononuclear cells from the previous donor were used. And he was given ruxolitinib (5 mg, qd) orally to prevent aGVHD. Day 24: the patient began to have a fever, accompanied by cough and expectoration. Chest CT (Figures 1(e) and 1(f)) showed bilateral patchy shadows and filamentous shadows in the lungs, and ground-glass opacity of the lower lobes was increased. From the perspective of lung CT, the pulmonary infection of the patient was more serious than before, and combined with the epidemic situation of COVID-19 in China at that time, SARS-CoV-2 infection was considered. Day 27: the SARS-CoV-2 nucleic acid test was positive, and he was transferred to a COVID-19-designated hospital.

**Figure 2:**
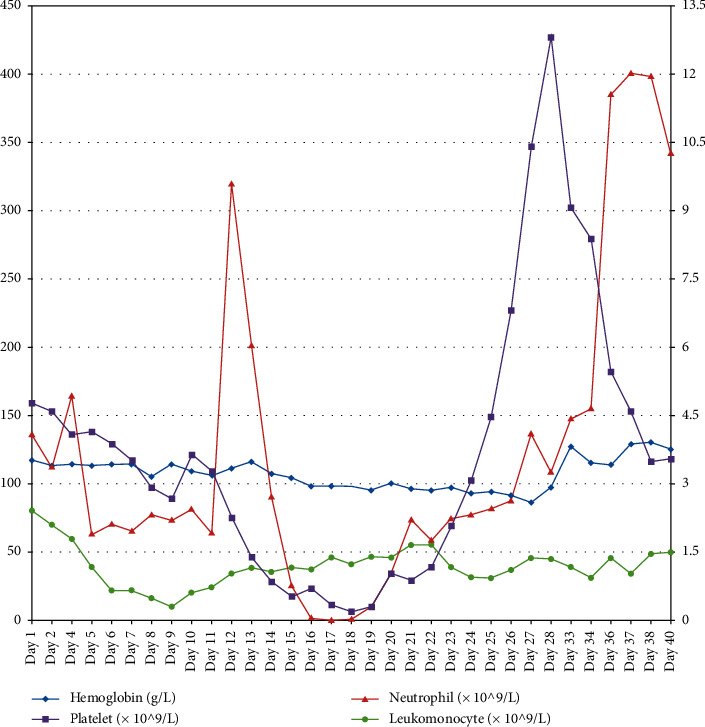
Changes of blood routine of the patient during hospitalization.

Day 28: the patient still had fever, shortness of breath, cough, and expectoration. His temperature was 38.7°C, blood pressure was 100/60 mmHg, heart rate was 106 beats per minute, respiratory rate was 23 breaths per minute, and oxygen saturation was 98% (nasal catheter oxygen inhalation 3 L/min). Bilateral wet rales could be heard in the lungs. The patient was given oxygen via nasal cannula, and drug treatment included arbidol (0.2 g, tid), mucosolvan (30 mg, qd), and magnesium isoglycyrrhizinate (100 mg, qd). Day 31: he developed facial and limb rashes (covering 50% of body surface area) and diarrhoea (approximately 1100 mL/d). Degree II-III aGVHD of the skin and gastrointestinal tract was considered because he had received haematopoietic stem cell infusions 20 days prior and stopped taking ruxolitinib on his own after the transfer. Considering that SARS-CoV-2 was highly contagious and had a high risk of infection during invasive operation, colonoscopy and skin biopsy were not performed. He was given ruxolitinib (5 mg, qd) orally again and methylprednisolone (20 mg, qd) intravenously (because he had fever in the early morning, he had been treated with 10 mg dexamethasone temporarily; therefore, we only used 20 mg methylprednisolone at the beginning). Day 32: SARS-CoV-2 nucleic acid test was still positive. Thus, arbidol was stopped, and he participated in the clinical trial of remdesivir. It was given at 200 mg intravenously on the first day and at 100 mg over the following 9 days. Methylprednisolone was increased to 40 mg qd. Day 34: his temperature fluctuated at 38–39°C. Blood bacterial and fungal cultures were negative, but C-reactive protein (CRP) increased to 119.10 mg/L. Meropenem (1 g, tid) was used to resist bacterial infection, and methylprednisolone was increased to 40 mg bid. Day 37: his aGVHD achieved partial remission (PR), and CRP had decreased to 36.70 mg/L. Then, he was transferred to the respiratory ICU ward for the unified management of patients in the remdesivir clinical trial. He was treated with remdesivir (100 mg, qd), cefoperazone sodium/sulbactam sodium (3 g, tid), linezolid (0.6 g, bid), methylprednisolone (40 mg, qd), and thymalfasin (1.6 mg/d). Day 38: his blood pressure dropped rapidly, and his neutrophil count was 11.96 × 10^9^/L, lymphocyte count was 1.45 × 10^9^/L (Figure 2), and procalcitonin was 47.87 ng/mL. Septic shock was considered. Noradrenaline was given immediately, and respiratory support was strengthened (nasal catheter oxygen inhalation was switched to high-flow oxygen inhalation through the nose). Day 39: he developed liver and kidney function injury and coagulation dysfunction. Day 41: he died of septic shock and multiple organ failure.

## 3. Discussion

When transplant recipients are infected with SARS-CoV-2, due to immune deficiency or delayed recovery of immune function, the virulence of the virus is stronger, and the viral clearance time is longer [3]. Therefore, posttransplant COVID-19 patients should actively receive antiviral therapy.

The pathogenic mechanism of SARS-CoV-2 has not been fully elucidated. Cao Bin found that there are higher levels of inflammatory cytokines in the plasma of critical COVID-19 patients than in noncritical patients, suggesting that cytokine storm is associated with the occurrence and severity of COVID-19 [4]. Therefore, anti-inflammatory therapy may play an important role in the treatment of COVID-19.

Corticosteroids are the most commonly used anti-inflammatory drugs, but their use has been controversial. Corticosteroids can inhibit excessive inflammatory responses and reduce the bodily damage caused by the inflammatory cytokine storm, but they can also prolong the viral clearance time and increase the risk of secondary infection. However, excessive inflammation and pulmonary damage caused by inflammatory cytokines may cause rapid deterioration due to the disease in critical COVID-19 patients. Compared to patients who only have COVID-19, this patient also had aGVHD; therefore, corticosteroids were used more actively.

In addition to corticosteroids, some immunomodulatory drugs are gradually undergoing trial usage. Ruxolitinib is a selective Janus kinase (JAK) 1/2 inhibitor that has been approved for use by the FDA in the treatment of steroid-refractory aGVHD after allogeneic haematopoietic stem cell transplantation [5]. Given that traditional immunosuppressants, such as calcineurin inhibitors, may increase the risk of infection and tumour recurrence, we used ruxolitinib to prevent and treat aGVHD in this patient. Ruxolitinib can not only prevent aGVHD but also retain the graft-versus-leukemia effect [6]. In addition, ruxolitinib also has an anti-inflammatory effect and has been applied to inflammatory cytokine storm and haemophagocytic syndrome [7]. For this patient, there was no severe respiratory distress in the initial stage of SARS-CoV-2 infection. In the later stage, the disease condition became complicated and serious due to aGVHD, but PR of aGVHD was achieved within a week, and the pulmonary infection was also quickly controlled. This suggests that appropriate immunosuppression in posttransplantation patients, as well as ruxolitinib combined with corticosteroid therapy for aGVHD, may help to alleviate and combat the excessive inflammatory cytokine storm caused by SARS-CoV-2.

The patient deteriorated rapidly after being transferred to another department, which may be related to the following factors: (1) Excessive inflammatory response, immune deficiency, and the use of corticosteroids led to low immune function and kept the patient under constant threat of various pathogens. (2) The ability of SARS-CoV-2 to infect the mucous membrane of the respiratory tract and digestive tract is very prominent [8, 9], and GVHD also easily attacks multiple organs and the vascular endothelium [10, 11]. The destruction of these natural physical and immune barriers greatly increases the risk of infection and mortality. (3) Premature reduction of corticosteroids and the application of immunomodulators after the patient was transferred to the pneumology department may have stimulated the intensity of the inflammatory reaction or the degree of autoimmune attack, resulting in the rapid deterioration of the disease. (4) When the intestinal and respiratory mucous membranes are destroyed or repaired incompletely, the production of ectopic or drug-resistant bacteria in the intestinal tract can easily lead to septic shock.

## 4. Conclusion

The disease condition of this COVID-19 patient was serious, complex, and variable due to aGVHD. However, there was no uncontrollable trend in progressive deterioration in the early stage, reflecting the importance of the balance between viral pathogenicity, moderate immune response, supportive therapy, and new small molecule drugs with anti-inflammatory and anti-GVHD effects. However, this patient eventually died of septic shock and multiple organ failure, suggesting that it is easy for new complications to arise in the treatment process of COVID-19 with aGVHD. Even if the disease improves, clinicians should be aware of changes in the disease and strengthen the prevention and treatment of infection to avoid life-threatening accidents or the deterioration of the disease.

## Figures and Tables

**Table 1:** Laboratory test results of the patient during hospitalization.

Measure	Reference range	Day 2 baseline	Day 24	Day 27	Day 28	Day 32	Day 37	Day 39
WBC (×10^9^/L)	3.5–9.5	7.45	3.54	5.94	5.22	6.33	13.99	12
NEUT (%)	40%–75%	54.9	89.5	69	62.4	70	85.9	85.5
NEUT (×10^9^/L)	1.8–6.3	4.09	2.46	4.1	3.26	4.43	12.02	10.26
LYM (%)	20%–50%	32.2	26	22.9	25.7	18.4	7.3	12.4
LYM (×10^9^/L)	1.1–3.2	2.4	0.92	1.36	1.34	1.16	1.02	1.49
Hb (g/L)	130–175	117	94	86	97	127	129	125
PLT (×10^9^/L)	125–350	159	149	347	427	302	153	118
D-dimer (mg/L)	0–0.5	0.62	—	1.65	2.13	1.73	—	6.69
FIB (g/L)	2–4	5.15	—	4.17	4.86	4.4	3,2	4.96
PT (s)	11–16	13.5	—	14.6	14.3	12.2	14.7	64.7
APTT (s)	27–45	53.1	—	49.3	56.9	41.7	36.9	57.9
ALT (U/L)	5–40	36	23	51	215	150	53	376
AST (U/L)	8–40	30	20	37	190	86	25	375
ALB (g/L)	33–55	34.7	39.4	36.2	39.4	22	26.2	23.4
T-bil (umol/L)	3–20	5.3	4.4	3.9	7	11.4	15.3	12.1
D-bil (umol/L)	1.7–6.8	0	2.2	2	4.5	6.7	7.8	8.1
BUN (mmol/L)	2.9–8.2	5.96	3.84	5.84	6.68	9.98	16.71	31.3
SCr (umol/L)	57–111	73	79.5	72.2	74.5	58.8	112.1	266.8
K^+^ (mmol/L)	3.5–5.3	3.72	4.17	4.25	5.85	3.92	4.01	5.09
Na^+^ (mmol/L)	137–147	139	136.9	133.8	130.6	129.2	140.4	133
CRP (mg/L)	<8	28.7	32.7	13.9	60.8	79.78	36.7	93.36
PCT (ng/mL)	<0.05	<0.13	0.37	0.52	10.18	1.48	—	47.87
IL-2 (pg/mL)	0.1–4.1	—	—	—	—	—	—	2.01
IL-4 (pg/mL)	0.1–3.2	—	—	—	—	—	—	1.07
IL-6 (pg/mL)	0.1–2.9	—	—	—	—	—	—	443.46
IL-10 (pg/mL)	0.1–5	—	—	—	—	—	—	4.39
TNF-*α*	0.1–23	—	—	—	—	—	—	1.2
IFN-*γ*	0.1–18	—	—	—	—	—	—	0.94

## Data Availability

No data were used to support the findings of this study.
